# Hemodynamic Functions of Fenestrated Stent Graft under Resting, Hypertension, and Exercise Conditions

**DOI:** 10.3389/fsurg.2016.00035

**Published:** 2016-06-14

**Authors:** Harkamaljot Singh Kandail, Mohamad Hamady, Xiao Yun Xu

**Affiliations:** ^1^Department of Chemical Engineering, Imperial College London, London, UK; ^2^Department of Interventional Radiology, St Mary’s Hospital, Imperial College Healthcare NHS Trust, London, UK

**Keywords:** endovascular aneurysm repair, fenestrated stent graft, rest, hypertension, exercise, hemodynamics, computational fluid dynamics

## Abstract

The aim of this study was to assess the hemodynamic performance of a patient-specific fenestrated stent graft (FSG) under different physiological conditions, including normal resting, hypertension, and hypertension with moderate lower limb exercise. A patient-specific FSG model was constructed from computed tomography images and was discretized into a fine unstructured mesh comprising tetrahedral and prism elements. Blood flow was simulated using Navier–Stokes equations, and physiologically realistic boundary conditions were utilized to yield clinically relevant results. For a given cycle-averaged inflow of 2.08 L/min at normal resting and hypertension conditions, approximately 25% of flow was channeled into each renal artery. When hypertension was combined with exercise, the cycle-averaged inflow increased to 6.39 L/min but only 6.29% of this was channeled into each renal artery, which led to a 438.46% increase in the iliac flow. For all the simulated scenarios and throughout the cardiac cycle, the instantaneous flow streamlines in the FSG were well organized without any notable flow recirculation. This well-organized flow led to low values of endothelial cell activation potential, which is a hemodynamic metric used to identify regions at risk of thrombosis. The displacement forces acting on the FSG varied with the physiological conditions, and the cycle-averaged displacement force at normal rest, hypertension, and hypertension with exercise was 6.46, 8.77, and 8.99 N, respectively. The numerical results from this study suggest that the analyzed FSG can maintain sufficient blood perfusion to the end organs at all the simulated conditions. Even though the FSG was found to have a low risk of thrombosis at rest and hypertension, this risk can be reduced even further with moderate lower limb exercise.

## Introduction

Until 1991, highly invasive open surgical repair (OSR) was the only standard treatment option for abdominal aortic aneurysms (AAA); however, with the advent of minimally invasive endovascular aneurysm repair (EVAR), the landscape of AAA treatment has changed drastically (1). EVAR involves fluoroscopically guiding and deploying a stent graft at the site of the aneurysm sac, and once deployed, this stent graft forms an artificial conduit for blood flow, thus alleviating AAA walls from adverse hemodynamic loads and preventing its rupture. As of 2005, EVAR has become the standard treatment procedure for infrarenal AAA not only because it is minimally invasive but also because it is associated with low mortality and morbidity rates along with shorter hospital stay (2–5). Stroupe et al. also found EVAR to be a more economical option for AAA repair than OSR (6).

Given these benefits of EVAR over OSR, until recently, a significant cohort of AAA patients, between 40 and 50%, were deemed unsuitable for this minimally invasive procedure either due to anatomical complications such as short and/or conical infrarenal aortic neck or extension of the AAA sac above the level of renal arteries (suprarenal AAA). Fenestrated stent grafts (FSGs) were developed specifically to overcome this limitation of EVAR and to make endovascular repair possible for this group of patients who were previously excluded from the EVAR envelope. FSGs rely on suprarenal fixation, and to preserve the indispensable renal arteries, separate bridging stent grafts known as renal branches are deployed in each renal artery through wire-reinforced holes or fenestrations in the main aortic body of the FSG (7). Once deployed, the FSG is designed to fully exclude the aneurysm sac from any blood flow while maintaining sufficient renal perfusion. These fenestrated devices show promising short- to mid-term results; however, the question of long-term durability remains unanswered (8, 9).

Thanks to interdisciplinary collaborations between engineering and medicine, considerable research effort has been made on addressing the question of long-term durability of FSG by performing detailed analysis of blood flow inside post-EVAR FSG using novel numerical techniques (10–16). Although many physiologically realistic geometric features of post-EVAR FSG, such as non-planarity, angulated aortic neck, and varying renal take-off angles, among others, have been examined in the past, previous numerical studies were mainly focused on the hemodynamic performance of FSG under normal resting conditions (10–16). Little is known about how this would change in response to hypertension or exercise. It is important to understand any changes in blood flow patterns and the associated hemodynamic forces because: (a) most of the AAA patients are older than 65 years who are hypertensive and (b) it is common that they are engaged in some level of physical activities on daily basis. Elucidating how the hemodynamic performance of FSG may change with varying physiological conditions will help us partly predict the durability of FSG.

The objective of this study was to elucidate the impact of hypertension and exercise on hemodynamic conditions in a patient-specific model of post-EVAR FSG by means of numerical simulation. To this end, we seek to quantify the following: (a) flow rate in the renal and iliac branches after EVAR, (b) detailed flow patterns within the FSG, (c) wall shear stress (WSS)-related indices, and (d) displacement forces.

## Methodology

Numerical simulations were performed on a patient-specific post-EVAR FSG using computational fluid dynamics (CFD). Performing CFD simulations is a systematic and multistep process that involves: (a) defining the 3D geometry describing the fluid domain, i.e., the FSG lumen, (b) discretizing this fluid domain into a fine computational mesh, and, finally, (c) solving the governing equations subject to appropriate boundary conditions. These steps are described in detail below.

### FSG Geometry

**Figure 1:**
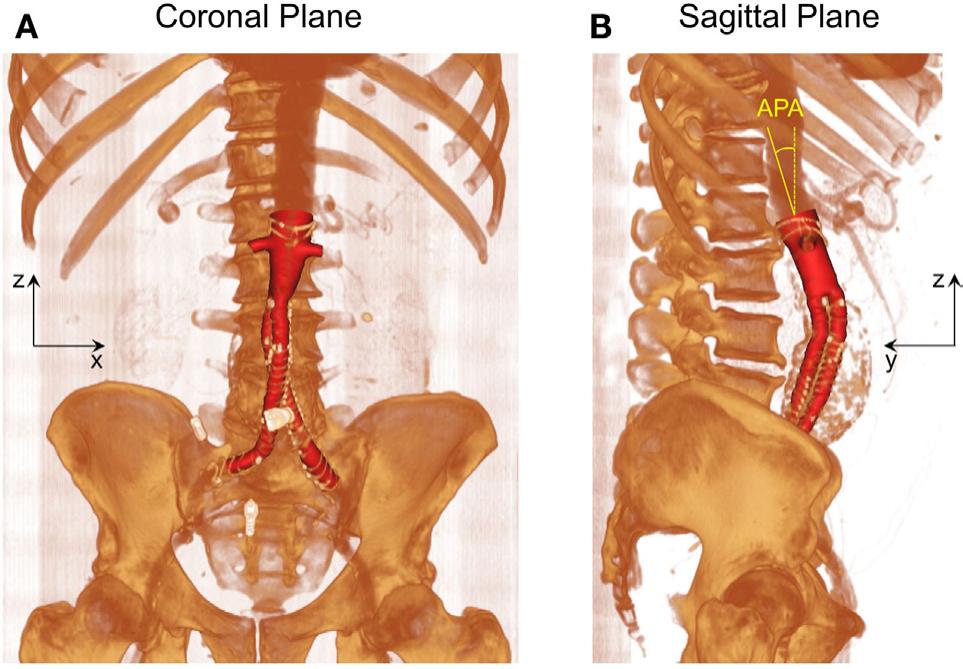
**Schematic of the fenestrated stent graft (FSG) geometry extracted from the computed tomography scans**. **(A)** Volume rendered image of the FSG in the coronal plane and **(B)** volume rendered image of the FSG in the sagittal plane with anterior/posterior angle (APA) clearly shown. APA is defined as the angle between the *z* axis (dashed yellow line) and the normal to the inlet (solid yellow line). Reprinted from Kandail et al. (36).

As shown in Figure 1, post-EVAR FSG geometry was extracted from contrast-enhanced computed tomography (CT) scans using a region growing algorithm available in Mimics (Materialise, Leuven, Belgium). The CT scans were obtained from St Mary’s Hospital, Paddington, London, and the data were analyzed anonymously. Internal institutional review board approval was not required for this limited and retrospective study. The inlet of the FSG was defined at the location where the proximal fixation hooks first appeared on the CT scan, whereas the outlets were identified when the circumferential metal rings of the FSG were no longer seen on the CT scan (15). Based on the terminology proposed by Molony et al., the segmented non-planar FSG had an anterior/posterior angle (APA) of 19.50° in the sagittal plane and a straight aortic neck (17). It is worth mentioning that the FSG geometry as shown in Figure 1 only comprises the lumen of the FSG, which represents the fluid domain of interest.

### Meshing the Fluid Domain

After the fluid domain was segmented from the CT images, it was then discretized into a fine unstructured mesh comprising tetrahedral and prismatic elements using ANSYS ICEM CFD (ANSYS Inc., Canonsburg, PA, USA). In CFD simulations, it is critical to resolve the boundary layer adequately, and for this reason, the near wall region was meshed with six layers of exponentially growing prism layers.

Mesh sensitivity studies were carried out, and the numerical results were declared mesh independent when the difference in time-averaged WSS (TAWSS) was <2% between two successive meshes. It was found that the minimum number of elements required to meet this requirement was 350,000. However, the final simulations were performed on an unstructured mesh with approximately one million elements since the computational time was not a major issue in this case.

### Governing Equations and the Boundary Conditions

Velocity and pressure values were obtained at every node of the computational mesh by numerically solving the Navier–Stokes equations using ANSYS CFX (ANSYS Inc., Canonsburg, PA, USA), which is a finite volume-based solver. In very simple terms, Navier–Stokes equations govern the mass and momentum conservation for blood flow, which is assumed to be incompressible (constant density of 1060 kg/m^3^), laminar, and Newtonian (dynamic viscosity of 0.004 Pa s). It is crucial to solve the Navier–Stokes equation at physiologically relevant boundary conditions in order to obtain clinically relevant results.

**Figure 2:**
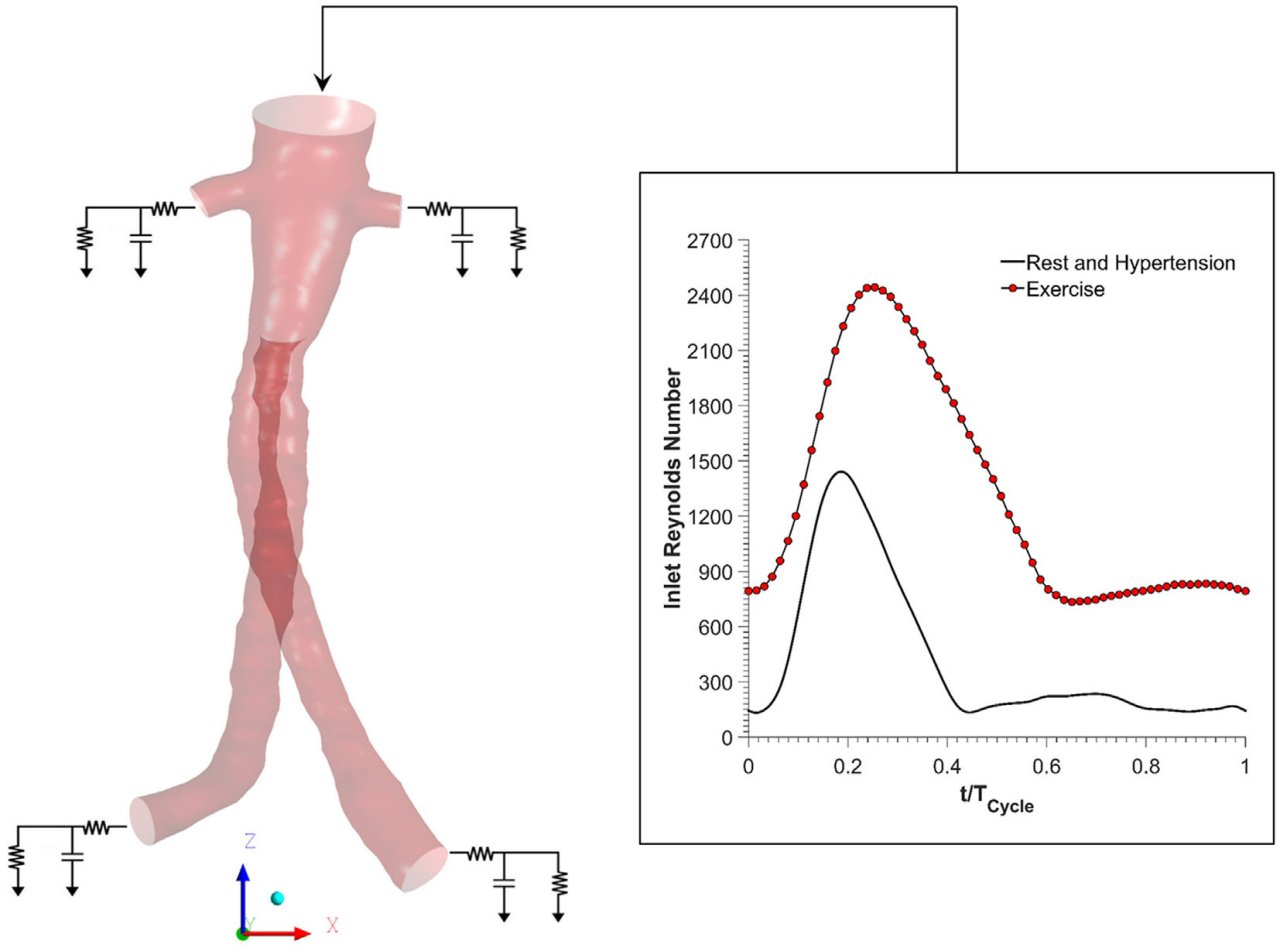
**Schematic of the numerical model used in the study**. Volumetric flow rate was imposed at the inlet, and this inflow waveform was adjusted accordingly for resting, hypertension, and exercise scenarios. No slip boundary conditions were imposed at the fenestrated stent graft walls, which were assumed to be rigid. Outlet pressure waveforms were obtained by coupling each outlet with a 3-element Windkessel model (3-EWM). Hypertension and hypertension plus exercise simulations were simulated by appropriately adjusting the parameters of the 3-EWM.

Figure 2 shows the boundary conditions employed in this study. To simulate resting conditions, a flow waveform typical of AAA patients at rest was imposed at the inlet along with Womersley velocity profiles, while no slip boundary conditions were prescribed at the FSG walls that were assumed to be rigid (18). Outlet pressure waveforms were obtained by coupling each outlet of the FSG with a 3-element Windkessel model (3-EWM), which represents the demands of the vasculature distal to FSG, and these outflow boundary conditions were implemented in ANSYS CFX through FORTRAN user subroutines. Based on the clinical data reported by Sonesson et al., the parameters of the 3-EWM were fine tuned to achieve resting systolic and diastolic aortic blood pressures of 130/60 mmHg (19).

Hypertensive conditions were simulated by increasing the peripheral resistance of the downstream vasculature in order to achieve higher systolic and diastolic aortic blood pressures of 170/90 mmHg, as reported by Montain et al. (20). Mayet and Hughes reported that elevation of blood pressure in hypertension was primarily due to increased peripheral resistance while the cardiac output remained about the same; therefore, the inflow waveform employed in hypertensive simulations was exactly the same as that of the resting conditions (21).

In order to simulate exercise conditions, both the inflow waveform and the parameters of the 3-EWM were adjusted accordingly using a methodology first proposed by Les et al. (18). Very briefly, both the heart rate and cardiac output were increased during moderate lower limb exercise. Meanwhile, the total resistance of the renal vasculature was increased, but the total resistance of the iliac vasculature was decreased in order to mimic the increased blood flow to the legs. It is worth mentioning that the exercise simulation was performed under combined hypertension and exercise condition in order to find out if moderate lower limb exercise can alleviate the adverse effects of hypertension. All the inflow waveforms employed in this study are summarized in Figure 2. A fixed time step of 0.001 s was adopted, and the convergence criterion based on the residual root mean square was set to be 1 × 10^−6^. CFD simulations were performed for four cardiac cycles in order to achieve periodicity, and the results presented in the succeeding section are from the final fourth cardiac cycle.

## Results

In order to systematically elucidate the hemodynamic performance of FSG under varying physiological conditions, the results are presented and analyzed according to the following subcategories: (a) renal and iliac outflow waveforms, (b) detailed comparison of flow patterns in the renal branches and the suprailiac bifurcation, (c) WSS-related indices, and (d) displacement forces acting on the FSG.

### Renal and Iliac Outflow Waveforms

**Figure 3:**
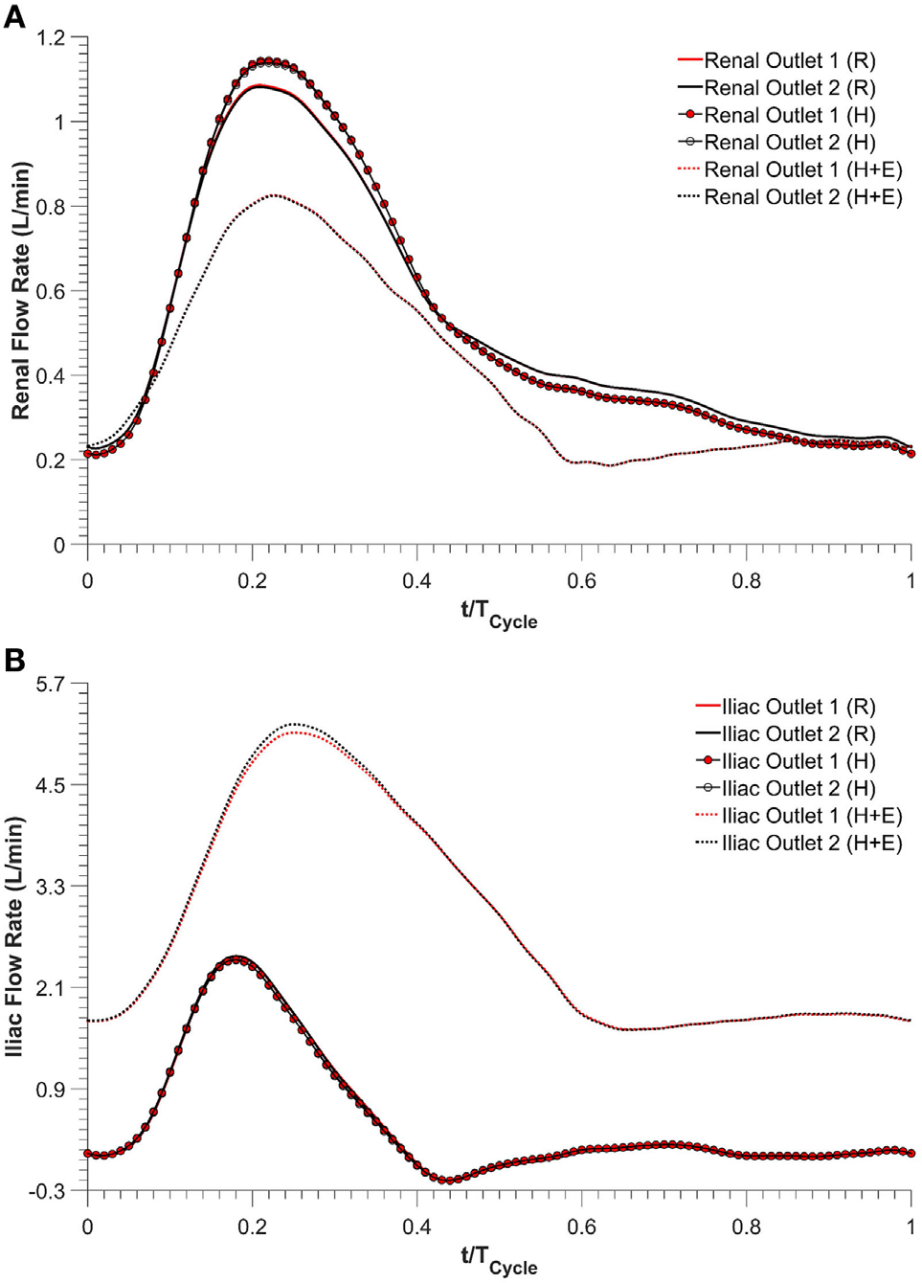
**Outflow waveforms, (A) renal outflow waveform and (B) iliac outflow waveform**. R refers to the resting conditions, H refers to the hypertension simulations, while H + E refers to exercise conditions simulated for hypertensive subjects.

Renal and iliac outflow waveforms are shown in Figure 3, and it can be observed that the flow split between the left and right renal arteries was identical for all the simulated physiological conditions. For cycle-averaged inflow of 2.08 L/min during rest, the percentage of the inflow that was channeled into the left and right renal branches was 24.90 and 24.86%, respectively. For the same cycle-averaged inflow at hypertension conditions, the percentage flow split into the renal branches was 24.84 and 24.82%, respectively. When hypertension was combined with moderate lower limb exercise, the cycle-averaged inflow was increased by 3.07 times to 6.39 L/min, and the flow split into each renal branch was only 6.29% of the cycle-averaged inflow. As a result, the cycle-averaged iliac flow increased significantly by 438.46% from resting conditions to 2.80 L/min in each iliac branch. The percentage flow splits into the individual renal and iliac outlets are summarized in Table 1. It can also be observed from Figure 3 that the flow in the renal arteries was antegrade during the entire cardiac cycle for all the simulated physiological conditions. In the iliac arteries, however, there was some flow reversal during early diastole at rest and hypertension, but the iliac flow was totally antegrade under moderate lower limb exercise conditions.

**Table 1:** **Percentage of the cycle-averaged inflow that is channeled into each renal and iliac outlets**.

	Percentage flow split (%)			
	Renal outlet 1	Renal outlet 2	Iliac outlet 1	Iliac outlet 2
Rest	24.90	24.86	25.11	25.13
Hypertension	24.84	24.82	25.16	25.18
Hypertension + exercise	6.29	6.29	43.56	43.86

*Cycle-averaged inflow during rest and hypertension was 2.08 L/min while cycle-averaged inflow during hypertension plus exercise was 6.39 L/min. It is worth mentioning that the inflow here refers to the infra-superior mesenteric arterial blood flow*.

### Comparison of Flow Patterns in the Main Stent Graft Endoprosthesis

**Figure 4:**
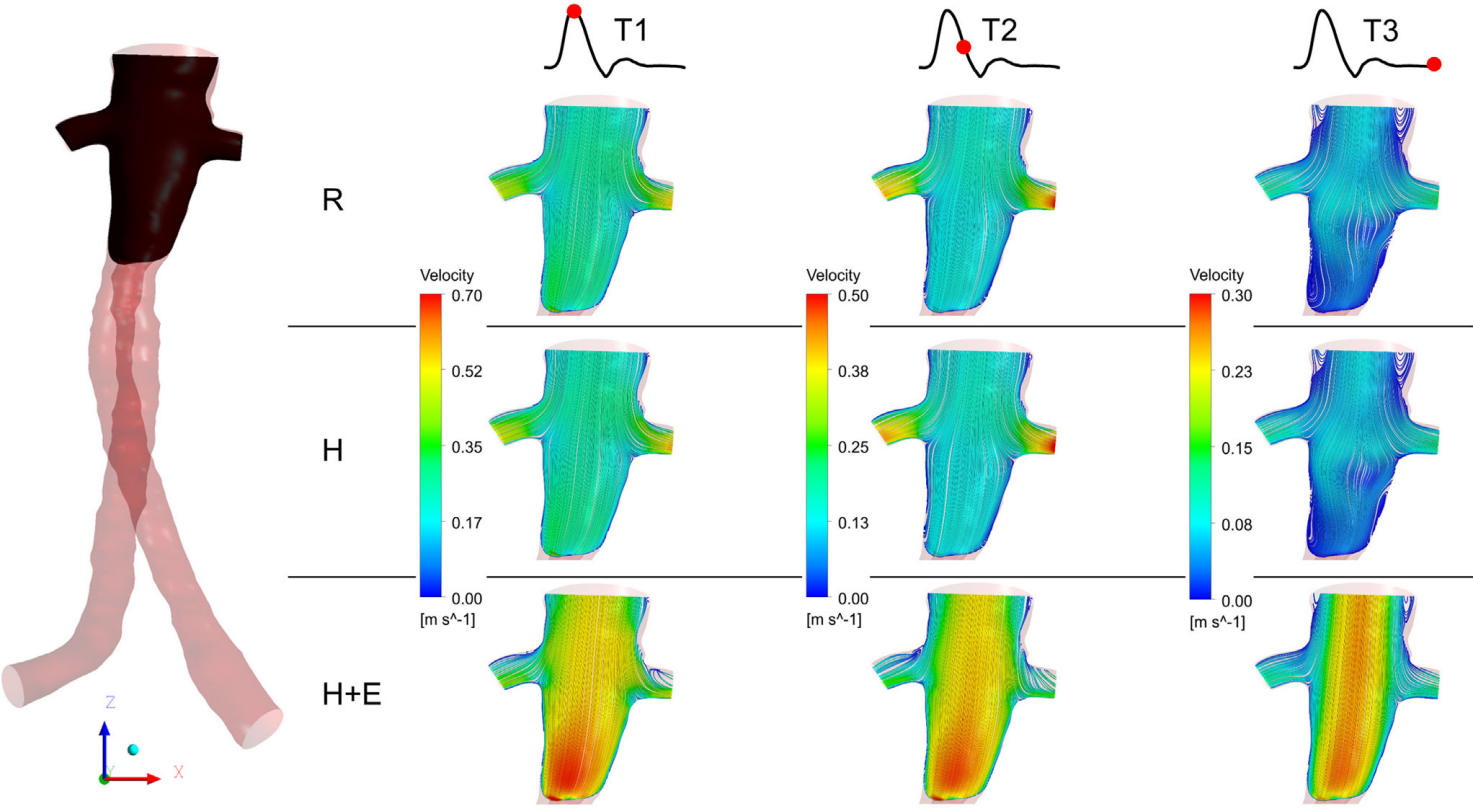
**Instantaneous velocity streamlines plotted at a cut plane in the coronal plane during T1, T2, and T3**. The location of the cut plane is shown in the fenestrated stent graft on the left. R refers to the resting conditions, H refers to the hypertension simulations, while H + E refers to exercise conditions simulated for hypertensive subjects. It can be noticed that the velocity streamlines throughout the entire cardiac cycle during resting and hypertension scenarios are very similar to one another, while the highest flow velocity was observed during the exercising conditions.

Three characteristic time points in the cardiac cycle were selected to elaborate instantaneous flow patterns in the main stent graft endoprosthesis and renal branches of the FSG (Figure 4) and these time points were (a) T1 – peak systole, (b) T2 – maximum flow deceleration, and (c) T3 – terminal diastole. It can be noticed from Figure 4 that at T1 and T2, flow streamlines within the main stent graft endoprosthesis were very well organized for all the physiological conditions. However, regions of disturbed flow were observed in the renal branches under exercise condition. At T3 under exercise condition, flow streamlines in the main stent graft endoprosthesis and the renal branches of the FSG were laminar with no flow recirculation; however, for resting and hypertensive conditions, two flow recirculation zones were observed at the suprailiac bifurcation. Figure 4 also shows that the flow patterns at resting and hypertensive conditions were very similar to one another, while the highest local flow velocities were observed under exercise conditions.

### WSS-Related Indices

**Figure 5:**
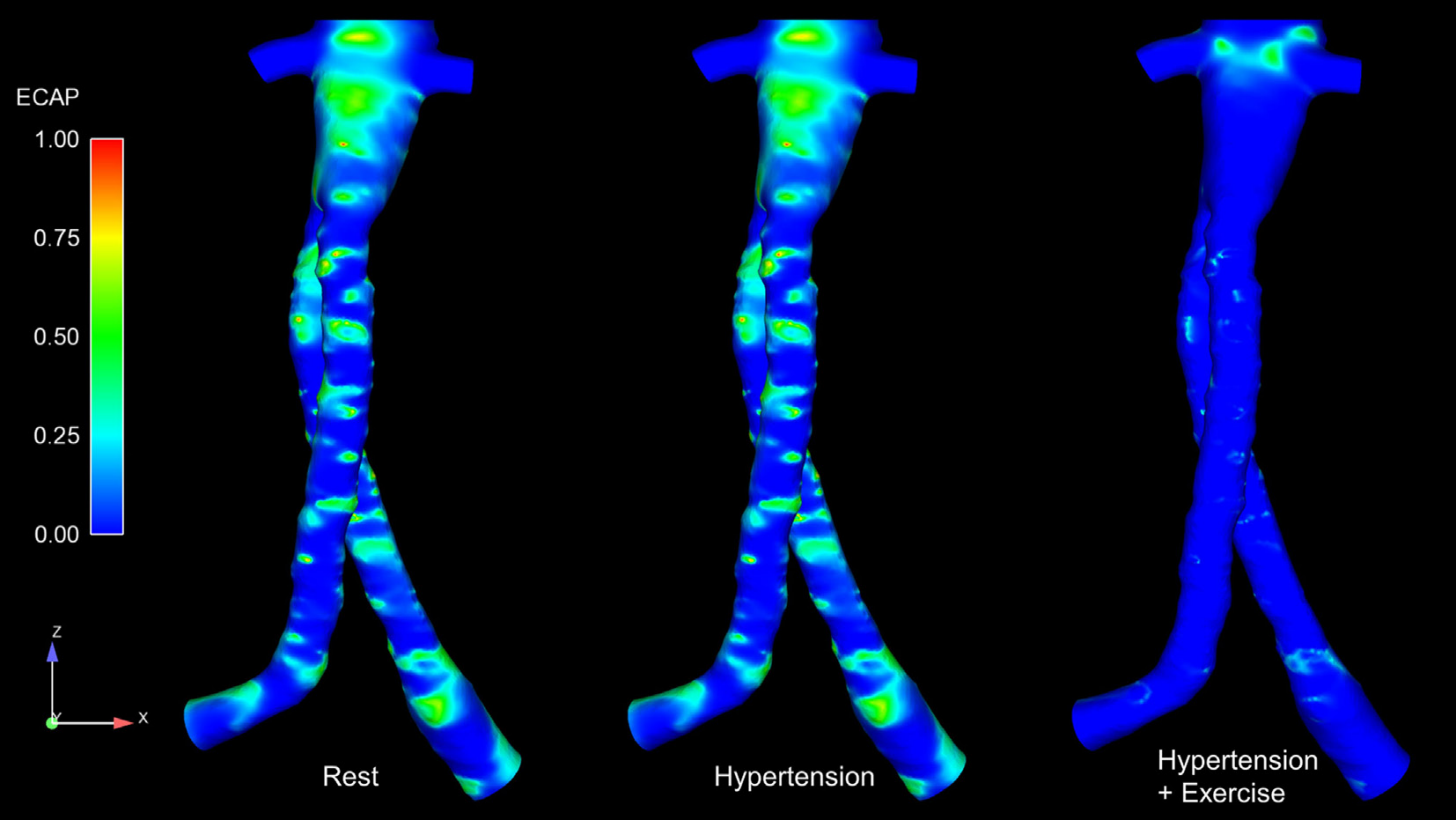
**Endothelial cell activation potential (ECAP) contours for all the analyzed physiological conditions**. These ECAP contours are shown in the coronal plane. It can be noticed clearly that the ECAP magnitude decreases during exercise conditions.

Regions exposed to high oscillatory shear index (OSI) and/or low TAWSS are known to favor cell adhesion and thrombus formation (22–24). Endothelial cell activation potential (ECAP), defined as the ratio of OSI to normalized TAWSS (25), can highlight regions simultaneously exposed to low TAWSS and high OSI. Di Achille et al. observed that within AAA, regions where ECAP >5.00 correlated positively with regions of thrombus formation (25). ECAP values were calculated for the FSG under rest, hypertension, and exercise conditions, and these are summarized in Figure 5. It is worth noting that the proximal part of the FSG between the inlet and the level of renal branches was excluded when quantifying ECAP values in order to eliminate the influence of artificially imposed inlet velocity profiles. Figure 5 shows clearly that ECAP values for the FSG are well below the reported “thrombosis-promoting” ECAP value of 5.00. ECAP values were further reduced during exercise, with the maximum ECAP values being 1.12, 1.12, and 0.69, for rest, hypertension, and exercise conditions, respectively.

### Displacement Forces Acting on the FSG

**Figure 6:**
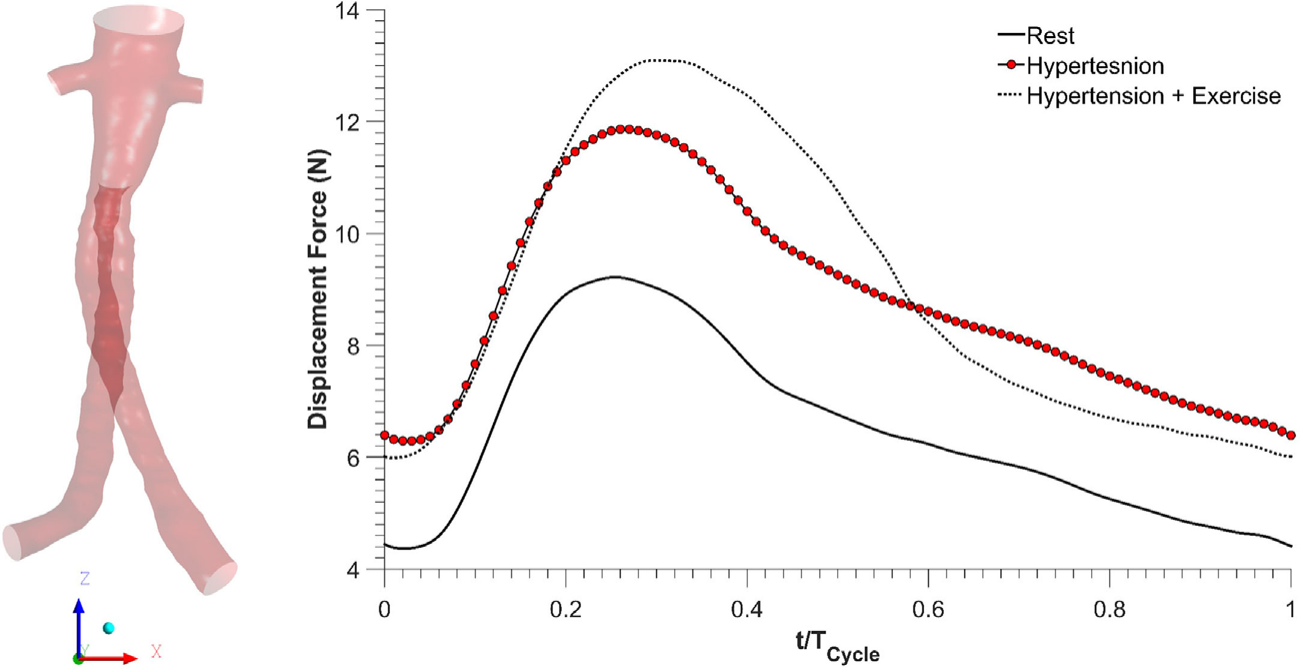
**Displacement forces acting on the fenestrated stent graft during all the simulated physiological scenarios**.

As blood flows through the FSG, it produces displacement forces on the FSG (26, 27), which can be evaluated by integrating pressure and WSS on the FSG wall. Figure 6 shows the variation in displacement forces over a cardiac cycle. It can be seen that the time dependence of displacement forces resemble the pressure waveforms very closely since WSS was negligible compared to pressure. The magnitude of displacement force depends strongly on the physiological condition, and its cycle-averaged value for rest, hypertension, and hypertension with exercise was 6.46, 8.77, and 8.99 N, respectively. The minimum, maximum, and cycle-averaged displacement forces experienced by the FSG for all the simulated physiological conditions are summarized in Table 2. The direction of the displacement force did not vary with the physiological and hemodynamic conditions and was dependent only on the device morphology. The displacement forces acting on the FSG presented here had an angle of 92°, 123.62°, and 144.38° with *x*, *y*, and *z* axis, respectively, as shown in Figure 6.

**Table 2:** **Minimum, maximum, and cycle-averaged (mean) displacement force experienced by the FSG**.

	Displacement force (N)		
	Minimum	Maximum	Mean
Rest	4.37	9.22	6.46
Hypertension	6.29	11.87	8.77
Hypertension + exercise	5.99	13.10	8.99

## Discussion

Fenestrated stent grafts were introduced to make EVAR possible for patients with complex AAA where there was insufficient length of healthy aortic neck below the renal arteries to act as a landing zone for the proximal part of the stent graft. FSGs rely on suprarenal fixation and maintain blood perfusion to the visceral side branches (e.g., renal arteries) through additional bridging stent grafts that are deployed into each side branch through the fenestrations within the main stent graft endoprosthesis. These fenestrated devices show promising short- to mid-term results, and the aim of this study was to assess their hemodynamic performance under different physiological conditions, namely, rest, hypertension, and hypertension with exercise.

Kidneys perform various physiological functions, and as such, they have a high demand on blood perfusion. It is estimated that, under normal resting conditions, between 15 and 30% of the infra-superior mesenteric arterial flow is channeled into each renal artery (28, 29). Based on these reference values, numerical results from this CFD study indicate that the simulated FSG can maintain sufficient blood perfusion to the kidneys during resting conditions as approximately 25% of the inflow was diverted into each renal artery (Table 1). As mentioned before in the Section “Methodology,” hypertension conditions were simulated by increasing the peripheral resistance of the downstream vasculature to achieve systolic and diastolic aortic blood pressures of 170/90 mmHg, while the inflow conditions were kept identical to the resting scenario. Increased peripheral resistance, as manifested through hypertension, had a negligible effect on renal perfusion as cycle-averaged flow into each renal artery was comparable to that under resting conditions (Table 1; Figure 3).

Exercise conditions were simulated in conjunction with hypertension to determine its effect on renal perfusion. As shown in Table 1 and Figure 3, during moderate lower limb exercise, the renal flow decreased (6.29% of the inflow), while iliac flow increased (43.56% of the inflow), and these observations are consistent with increased blood flow to the legs based on these conditions. In their *in vivo* study, Cheng et al. found that, during moderate lower limb exercise, the infra-renal flow in healthy subjects increased by 444.40% from 0.90 to 4.90 L/min (30), and similar results were also observed by Les et al. and Tang et al. (18, 31). The CFD results obtained in this study showed that the cycle-averaged infra-renal flow during rest (and hypertension) was 1.04 L/min, which increased to 5.59 L/min during exercise, i.e., an increase of 437.50%, which is in agreement with the *in vivo* findings of Cheng et al. Due to the characteristically low peripheral resistance of the kidneys, the renal flow waveform is always antegrade during the entire cardiac cycle (32) and as shown in Figure 3, the renal flow waveform unlike iliac flow waveform had no flow reversal. Based on these results, it can be deduced that FSG implantation for AAA repair does not adversely affect the flow to the renal and iliac arteries and can maintain sufficient blood perfusion to the end organs under different physiological conditions.

Figure 4 summarizes the instantaneous flow and velocity patterns in the main stent graft endoprosthesis and the renal branches at different time points (T1, T2, and T3) in the cardiac cycle. It can be clearly seen that with FSG in place, the flow is very well organized throughout the cardiac cycle without notable flow recirculation. Under resting and hypertension conditions, the absence of significant flow recirculation zones in the FSG led to high TAWSS and low OSI values. Regions of the FSG, which might be at potential risk for thrombosis, were highlighted through ECAP values, and due to high TAWSS and low OSI, the resulting ECAP values were low in the entire FSG (the maximum ECAP value at rest and hypertension was 1.12). As mentioned before in the Section “Results,” these low ECAP values suggest that the examined FSG is at low risk of thrombus formation based on the findings of Di Achille et al. (25). The ECAP contours shown in Figure 5 show little difference between resting and hypertension conditions.

Due to the significantly increased inflow and therefore velocities, the TAWSS values increased and OSI values decreased even further during moderate lower limb exercise as compared to the resting and hypertension conditions, and this led to even lower ECAP values in the FSG during exercise with a maximum ECAP of 0.69. These findings are consistent with the observations of Les et al., Cheng et al., and Tang et al., who also reported higher TAWSS and lower OSI under exercise conditions (18, 30, 31). Based on these flow features (Figure 4) and ECAP contours (Figure 5), it can be deduced that the analyzed FSG is at low risk of thrombosis, and with moderate levels of lower limb exercise, the risk of thrombosis can be reduced even further. At this point, it is worth clarifying that thrombus formation was not modeled explicitly in this study, but a hemodynamic metric was utilized to identify regions susceptible to thrombus formation using reported trends in the literature (this point is further elaborated in the Section “Limitations” below).

Apart from thrombosis, the other condition that affects the long-term durability of FSG is the displacement force acting on the FSG because it can potentially lead to device migration, which might necessitate secondary intervention (15–17, 26, 27). It can be seen from Figure 6 that hypertension and hypertension with exercise significantly increase the magnitude of displacement forces due to increased mean aortic blood pressure. It is also worth noting that when exercise was combined with hypertension, the peak displacement force was higher than that of the hypertension only scenario; however, the minimum displacement force during exercise was lower than that without exercise. This is because under dynamic exercise conditions, the systolic blood pressure increases, whereas the diastolic blood pressure decreases (18). Since the magnitude of the displacement force depends very strongly on the aortic pressure waveform, this change is also reflected in the displacement waveform. Cycle-averaged displacement forces for these physiological conditions are summarized in Table 2, and to put these values in perspective, they need to be compared with the experimental threshold values for device migration. Rahmani et al. analyzed various stent graft designs for EVAR and reported the effect of non-planarity (APA) on displacement forces required to dislocate the stent grafts (33). They reported that for a stent graft with an APA of 19.50°, the mean displacement force required to dislocate its proximal sealing section was approximately 32 N. Since the cycle-averaged displacement forces for the analyzed FSG under various physiological conditions are always well below this threshold value of 32 N, it can be deduced that this FSG is at low risk of migration.

## Limitations

First and most importantly, when the bridging renal stent grafts of FSG are deployed, a considerable section of the bridging stent grafts (between 3 and 6 mm) protrudes into the lumen of the main stent graft endoprosthesis. This protruded section of the bridging stent graft is then dilated considerably using a balloon to form a funnel-like shape in order to secure it in place and prevent its distal migration; this procedure is known as “flaring.” It is obvious and evident that flared renal stent grafts will disturb local blood flow and might lead to complex flow patterns including recirculation and swirling. However, these renal flares were not included in this study because due to the high density of the metal wires in the renal stent grafts, there were strong reflections in the CT images, making it impossible to distinguish the renal flares from lumen during image segmentation. Second, as mentioned earlier in the Section “Discussion,” thrombus formation was not modeled explicitly and its risk was assessed through ECAP, which is a hemodynamic index, and only highlights regions simultaneously exposed to high OSI and low TAWSS. ECAP does not consider the biochemistry associated with thrombus formation, such as platelet activation and the coagulation cascade, and these processes should be included in future studies for a more reliable prediction of the risk for thrombosis. Third, it was hypothesized that stent graft migration is dependent only on the magnitude and direction of the displacement forces. However, apart from displacement forces, there are other anatomical and biomechanical factors as well that contribute significantly to stent graft migration and failure, such as post-EVAR aneurysm shrinkage (34) and the complex mechanics of contact between FSG and aorta (35), to name a few. In its current form, the computational model utilized in this study cannot predict these additional complexities that also contribute considerably toward device migration. Fourth, the stent graft walls were assumed to be rigid rather than compliant; however, as the graft material from which these stent grafts are manufactured has extremely high Young’s moduli, the compliant walls are expected to have negligible effects on the presented results. Finally, only one FSG geometry was analyzed in this study. More FSG geometries with varying anatomical features should be examined in the future.

## Conclusion

Computational fluid dynamics simulations with physiologically realistic outflow boundary conditions can provide detailed insights into the post-EVAR hemodynamic performance of FSG. Based on the numerical results presented here, it can be concluded that the analyzed FSG can maintain sufficient renal and iliac blood perfusion under resting, hypertension, and hypertension combined with exercise. Due to the well-organized local flow feature and low ECAP values, the risk for thrombus formation in the simulated FSG is low, and moderate lower limb exercise can reduce the risk even further. Even though hypertension and exercise increase the magnitude of displacement forces, which are responsible for device migration, the maximum displacement force is well below the threshold to allow us to conclude that this FSG has a very low risk of migration.

## Author’s Note

Data supporting this publication can be obtained on request from biofluids-and-transport@imperial.ac.uk.

## Author Contributions

HK: conceived the study, performed all the CFD simulations, and drafted the manuscript. MH: conceived the study, provided the CT scan, and reviewed the manuscript. XX: conceived the study, provided guidance and resources to carry out the CFD simulations, and reviewed the manuscript.

## Conflict of Interest Statement

The authors declare that the research was conducted in the absence of any commercial or financial relationships that could be construed as a potential conflict of interest.
